# Combined cellomics and proteomics analysis reveals shared neuronal morphology and molecular pathway phenotypes for multiple schizophrenia risk genes

**DOI:** 10.1038/s41380-019-0436-y

**Published:** 2019-05-29

**Authors:** Martina Rosato, Sven Stringer, Titia Gebuis, Iryna Paliukhovich, Ka Wan Li, Danielle Posthuma, Patrick F. Sullivan, August B. Smit, Ronald E. van Kesteren

**Affiliations:** 1 Department of Molecular and Cellular Neurobiology, Center for Neurogenomics and Cognitive Research, Vrije Universiteit Amsterdam, Amsterdam, The Netherlands; 2 Department of Complex Trait Genetics, Center for Neurogenomics and Cognitive Research, Vrije Universiteit Amsterdam, Amsterdam, The Netherlands; 3 Department of Genetics, University of North Carolina, Chapel Hill, NC USA; 4 Department of Medical Epidemiology and Biostatistics, Karolinska Institutet, Stockholm, Sweden; 5 Department of Psychiatry, University of North Carolina, Chapel Hill, NC USA

**Keywords:** Neuroscience, Cell biology

## Abstract

An enigma in studies of neuropsychiatric disorders is how to translate polygenic risk into disease biology. For schizophrenia, where > 145 significant GWAS loci have been identified and only a few genes directly implicated, addressing this issue is a particular challenge. We used a combined cellomics and proteomics approach to show that polygenic risk can be disentangled by searching for shared neuronal morphology and cellular pathway phenotypes of candidate schizophrenia risk genes. We first performed an automated high-content cellular screen to characterize neuronal morphology phenotypes of 41 candidate schizophrenia risk genes. The transcription factors *Tcf4* and *Tbr1* and the RNA topoisomerase *Top3b* shared a neuronal phenotype marked by an early and progressive reduction in synapse numbers upon knockdown in mouse primary neuronal cultures. Proteomics analysis subsequently showed that these three genes converge onto the syntaxin-mediated neurotransmitter release pathway, which was previously implicated in schizophrenia, but for which genetic evidence was weak. We show that dysregulation of multiple proteins in this pathway may be due to the combined effects of schizophrenia risk genes *Tcf4*, *Tbr1*, and *Top3b*. Together, our data provide new biological functions for schizophrenia risk genes and support the idea that polygenic risk is the result of multiple small impacts on common neuronal signaling pathways.

## Introduction

Schizophrenia (SCZ) is a severe neuropsychiatric disorder characterized by persistent delusions and hallucination and abnormal social behavior. SCZ has a high heritability and genetic risk factors have an important role in disease pathogenesis [1, 2]. Despite many years of research, few genes have been identified that may be directly involved. Whole-exome sequencing led to the identification of *SETD1A* [3] and *RBM12* [4] and three rare copy number variants (CNVs) have been identified that impact single genes (i.e., *NRXN1* [5], *TOP3B* [6], and *VIPR2* [7]). Other CNVs have been identified [6, 8–11], but these are difficult to interpret functionally because they change the dosages of many genes and often present with complex, multi-system clinical phenotypes.

In contrast, common variation in the genome seems to have a large impact on the development of SCZ [2]. More than 145 independent genomic risk loci have been associated with SCZ or in genome-wide association studies (GWAS) [12, 13]. Some of these loci include associated gene variants that can modify the expression of large numbers of other genes or proteins. Examples include *miR137* [14] genes whose transcripts are bound by the fragile X mental retardation protein (FMRP) [15], and several transcription factors (e.g., *TCF4* [16]), providing an extra layer of genetic complexity to the disease. Together, all evidence suggests that SCZ is a highly polygenic disorder with many genes contributing to its development and symptomatology.

The high number of candidate risk loci poses an unprecedented biological challenge. It is currently not understood how this polygenic risk translates into common patterns of brain alterations underlying the disease. Are there many different cellular and molecular pathways that lead to disease, or do risk genes converge onto a small number of shared pathways underlying SCZ? We addressed this issue by performing a combination of cellular phenotyping (cellomics) and proteomics analysis. We used RNA interference in combination with automated high-content screening to identify neuronal phenotypes associated with reduced expression of 41 SCZ risk genes, and subsequent proteomics analysis to identify cellular pathways for genes sharing similar phenotypes. Our data show that inhibition of *Tcf4*, *Tbr1*, and *Top3b* each cause a similar synaptic morphological phenotype. The three genes converge onto a neurotransmitter release pathway that was previously associated with SCZ, and molecular changes induced by each gene are needed to significantly impact this pathway. Our data suggest that polygenic risk in SCZ may at least in part converge onto common cellular disease pathways that cannot be detected by computational pathway enrichment analyses alone, and that the high polygenic risk challenge in SCZ may be solved at the level of experimentally defined pathway sharing.

## Materials and methods

### Primary neuron culture

Hippocampal primary neuron cultures from E18 wildtype C57Bl/6 mouse embryos were prepared as previously described [17]. Hippocampal tissue was incubated 25 min at 37 °C in a Hanks balanced salt solution (Sigma) containing 1% HEPES buffer solution (1 m; Gibco) and 10% trypsin (Gibco). After three washes, the tissue was placed in Neurobasal medium (Gibco) completed with 2% B27 (Gibco), 2% HEPES solution, 0.25% glutamine (200 mm; Gibco) and 0.1% Pen/Strep (Gibco) and triturated with a fire-polished Pasteur pipette. Cells were counted in a Fuchs-Rosenthal chamber and plated in multi-well plates (Greiner Bio-one) that were previously coated with poly-d-lysine (Sigma). Cells were plated at 12.5K/well in 96-well glass bottom plates for morphological analyses, at 125K/well in 24-well plates for RNA extraction, or at 300K/well in 12-well plates for protein extraction.

### Lentivirus production

Bacterial glycerol stock (MISSION library, Sigma; Supplementary Table S1) were grown in agar plates with LB medium and 1% ampicillin. Single colonies were picked and expanded for DNA extraction (QIAprep spin mini prep kit; Qiagen). HEK 293T cells were transfected with the small hairpin RNA (shRNA) plasmid DNA together with envelope and packaging plasmids. One day after transfection medium was replaced with Optimem medium (Gibco) completed with 1% Pen/Strep and 1% glutamine. On the third day, the medium was collected and centrifuged at 1000 ×*g* for 5 min; the supernatant containing the viral particles was filtered (0.45 μm pore size) and aliquoted.

### Virus infection efficiency test

Primary hippocampal neurons were plated and infected at DIV1 with 1, 3, or 6 μl virus in 200 μl culture medium per well. At DIV2, puromycin (0.2 mg/ml; Gibco) was added. Cells were fixed at DIV7 and stained with Hoechst (1:10,000; Invitrogen) and anti-MAP2 (1:5000; Bio-connect). Infection efficiency was determined as the percentage of living cells compared with untreated non-infected control wells.

### Immunocytochemistry

Hippocampal neurons were infected with shRNAs at DIV1 and cultures were fixed and stained at DIV7, DIV14, or DIV21. Cells were fixed with 4% paraformaldehyde and 4% sucrose in phosphate-buffered salie (PBS; pH 7.4) followed by permeabilization with PBS containing 0.5% Triton X-100. Cells were incubated at room temperature (RT) with PBS containing 0.1% Triton X-100 and 1% BSA and then for two nights at 4 °C in PBS containing 0.1% Triton X-100, 1% BSA, anti-synapsin 1 (1:1000; Chemicon/Millipore: #AB1543P), anti-PSD-95 (1:250; Thermo Scientific; #MA1046) and anti-MAP2 (1:5000; Chemicon/Millipore, #AB5543). After washing twice with PBS, neurons were incubated for 90 min at RT in Alexa-488-conjugated goat anti-mouse (1:400; Abcam, #A11001), Alexa-568-conjugated goat anti-rabbit (1:400; Abcam, #A11011) and Alexa-647-conjugated goat anti-chicken (1:400; Abcam, #A21449). After washing twice with PBS and once with distilled H_2_O, the neurons were incubated for 10 min at RT with Hoechst (1:10000; Invitrogen).

### Imaging and image analysis

Images were acquired on an Opera™ LX (PerkinElmer) automated confocal microscopy system at × 10 and at × 40 magnification and were analyzed using Columbus image data storage and analysis software (v2.5.2.124862; PerkinElmer). Exposure and image analysis settings were kept constant throughout the entire screen. At × 10 magnification the entire well was imaged and quantitative parameters related to cell numbers and the length and complexity of dendritic trees were extracted based on Hoechst and anti-MAP2 staining. Glial nuclei were excluded based on their larger size and dimmer Hoechst staining compared with neurons and average MAP2 intensity per soma (i.e., in a circle around each nucleus) was used to select neurons. Dendrites were traced based on MAP2 staining. Dendrite selection parameters were set such that background MAP2 staining (often glial cells or debris) did not contribute to dendrite length measurements. Dendrite roots (primary dendrite origins) and nodes (branch points) were collected as measures of dendrite complexity. At × 40 magnification quantitative parameters related to synaptic development were extracted based on anti-MAP2, anti-synapsin and anti-PSD-95 staining. Synapsin-positive presynaptic puncta and PSD-95-positive postsynaptic puncta were traced specifically on MAP2-positive dendrites. Ten relevant measures of neuronal survival and network development were calculated: total number of nuclei per well, total number of neurons per well, number of primary neurites per neuron, number of dendritic branch points per neuron, total dendrite length per neuron, number of presynaptic puncta per dendrite length, presynaptic puncta average intensity, number of postsynaptic puncta per dendrite length, postsynaptic puncta average intensity, and number of colocalized pre- and postsynaptic puncta per dendrite length.

### Cellomics data analysis

Cellomics data (*n* = 3 cultures per shRNA) was normalized per plate against the scrambled control and log2 transformed. Log-normalized data were checked for normality of distribution and rank correlation was used to check for parameter correlation. Multi-level modeling was applied to the data to estimate the contribution of technical and experimental variation to the overall variance of each parameter. The R package lme4 [18] was used to fit a mixed-effect model for every parameter. Batch, plate, edge, DIV, and shRNA treatment were taken as potential sources of variation. A Mann–Whitney *U* test was performed per gene/parameter combination under the null hypothesis that the distribution of control wells and experimental wells behave the same. A null distribution of 10,000 *p* values was generated for each gene/parameter by a random shuffle of control and experimental well data within each plate. The Mann–Whitney *U* test was performed on this permutated parameter set per gene. The null-*p* value was then used to obtain an empirical *p* value by comparing the distribution of observed and null-*p* values. To correct for multiple testing, a second permutation analysis was performed and the minimum *p* value for all gene tests per permutated sample was acquired. Again, an empirical *p* value was obtained by comparing the observed and minimum *p* value distributions. This final empirical *p* value can be interpreted as the corrected *p* value and accounts for the number of tests as well as the correlation between test statistics. Cluster analysis of shRNA- or gene-level phenotypes was performed using a Pearson correlation distance matrix. Significance of shRNAs co-clustering was determined using a *χ*^2^-test.

### Real-time qPCR

RNA was extracted from hippocampal neurons at DIV7 (*n* = 4 cultures per shRNA) using the RNAeasy mini kit (Qiagen). RNA concentration was determined using the NanoDrop ND-1000 spectrophotometer (NanoDrop Technologies). For cDNA synthesis, 200 ng RNA was mixed with hexanucleotide primers (25 pmol/μl), heated to 37 °C for 1 min, and then snap-cooled on ice. A mix with reverse transcriptase (200 units/μl; Promega) and dNTPs (10 mm) was added and samples were incubated for 45 min at 37 °C. Real-time qPCR was performed using SYBR green (GC Biotech) as the reporter dye. The following primers were used: *Tcf4* (fwd: TGAACCCGGCAAACCCTGAA, rev: TCCCTAAGGCAGCCATTCGC), *Top3b* (fwd: GAGCCGCGTTTAGTGGGCA, rev: GCCAATGCTGACTCCTCGGG), *Tbr1* (fwd: GGCGGATCCCAATCACTGGA, rev: AGACCCGGTTTCCTTGCACA). *Hprt* (fwd: ATGGGAGGCCATCACATTGT, rev: ATGTAATCCAGCAGGTCAGCAA) and *Actb* (fwd: GCTCCTCCTGAGCGCAAG, rev: CATCTGCTGGAAGGTGGACA) were used for normalization. Data were analyzed using the 2^−ΔΔCp^ method [19].

### Proteomics

Proteins were extracted from neuronal cultures at DIV7 (*n* = 3 cultures per shRNA). After washing twice with PBS at 4 °C, 500 μl of PBS with protease inhibitor (Roche) was added to each well. Cells were scraped and recovered in Eppendorf tubes, centrifuged at 3000 × *g* for 5 min at 4 °C, supernatant discarded, and cells resuspended in 15 μl of loading buffer. Samples were processed for mass spectrometry as described previously [20, 21]. Protein samples were run on an sodium dodecyl sulfate polyacrylamide gel electrophoresis gel and the gel was fixed and stained with Coomassie blue. Gel lanes were cut into small pieces, de-stained with two incubations with 50 nm ammonium bicarbonate (Fluka)/50% acetonitrile (JT Baker) and dried with 100% acetonitrile. Proteins were in-gel digested overnight at 37 °C with trypsin (Promega) and the digested peptides were extracted with two incubations with 0.1% trifluoroacetic acid (Applied Biosystems)/50% acetonitrile and one incubation with 0.1% trifluoroacetic acid/80% acetonitrile. The eluted peptide solution was dried in a speedvac and dissolved in 0.1% acetic acid solution before being loading into an Ultimate 3000 liquid chromatography system (Dionex, Thermo Scientific). Peptides were electro-sprayed into the TripleTOF 5600 mass spectrometer (Sciex), with a micro-spray needle voltage of 5500 V and analyzed by data independent acquisition. Each SWATH cycle consisted of a parent ion scan of 150 msec and 8 Da SWATH windows, with scan time of 80 msec, through 450–770 m/z mass range. The collision energy for each window was calculated for a 2+ ion centered upon the window (spread of 15 eV).

### Proteomics data analysis

MS spectra were analyzed with Spectronaut software (Biognosys) and searched against a spectral library of cultured mouse primary hippocampal neurons for peptide identification. The optimal quality (*q*) value threshold for each proteomics data set was set on the average median *q* value of all samples. For protein identification, peptides were allowed to fail detection in only one replicate sample within groups and all but one samples between groups. Outlier analysis was performed when the sample coefficient of variance was higher than 0.12. In that case, replicates with median *q* values deviating > 10 times compared with other replicates were considered outliers and were removed from the data set. After removal of contaminant proteins (i.e., immunoglobulins, keratins, and trypsin), regulated proteins were selected based on two criteria: proteins had to show (i) a significant log2-fold change compared with scrambled control in at least one shRNA sample per gene (Student’s *t* test, *p* < 0.05), and (ii) a concordant log2-fold change (in the same direction, but not necessarily significant) in all other shRNA samples per gene. Protein–protein interaction analysis was performed using STRING [22]. “Interaction sources” was set to “experiments” only and “interaction score” was set to “high confidence” (0.7 or higher). Addition of a maximum of 10 1st shell interactors was allowed. Functional enrichment was tested for GO terms using gProfiler [23]. Enrichment was tested for “molecular function” GO terms only.

## Results

We selected 41 neuronally expressed genes for which an association with SCZ has been demonstrated or suggested (Table 1). Most genes (25) are genetically associated with SCZ, others are associated with autism spectrum disorder (ASD; 19 genes) or bipolar disorder (BPD; 10 genes) and have been implicated in SCZ otherwise. For instance, many predicted *miR137* and FMRP targets are included as these represent dysregulated pathways in SCZ [14, 15]. Full gene names, gene function summaries, and additional evidence for SCZ involvement for all genes are listed in Supplementary Table S2. For all genes, shRNA constructs were obtained for RNA interference. Given the variation in efficacy and the possibility of off-target effects, multiple shRNA constructs per gene were used (Supplementary Table S1). shRNA-containing lentiviral (LVV) particles were produced and tested for infection efficiency on mouse primary hippocampal neurons (Supplementary Fig. S1a). For each LVV production batch, a random selection of shRNAs was tested including at least one shRNA per target gene. At optimal virus concentrations, an average infection efficiency was observed of 80% ± 28% (Supplementary Fig. S1b). Real-time quantitative PCR demonstrated a knockdown efficiency of 64% ± 15% at 14 days post infection (*n* = 5; data not shown).

**Table 1:** List of selected genes and evidence for their association with SCZ, ASD of BPD

Gene	SCZ evidence	ASD evidence	BPD evidence
*Adnp*	Exome sequencing [44], FMRP target [45, 46]	De novo mutation [39]	
*Ank2*	FMRP target [45, 46]	De novo mutation [39]	
*Arid1b*	FMRP target [45, 46]	SNP [47], de novo mutation [39]	SNP [48]
*Atp2a2*	GWAS [12], FMRP target [45, 46]		
*Bcl11a*	miR137 target [14]		
*C4a*	GWAS [12]		
*Cacna1c*	GWAS [12]	SNP [49]	SNP [50]
*Cacna1d*	miR137 target [14]		SNP [51]
*Cacna1i*	GWAS [12], miR137 target [14], FMRP target [45, 46]	SNP [49]	
*Cacna2d3*	Exome sequencing [52]	De novo mutation [39]	
*Cacnb2*	GWAS [12], miR137 target [14]		SNP [53]
*Chd8*	De novo mutation [54], FMRP target [45, 46]	De novo mutation [39]	
*Cntnap2*	SNP [55]		SNP [55]
*Csmd1*	GWAS [12], miR137 target [14]		SNP [56]
*Cttnbp2*	miR137 target [14]		
*Drd2*	GWAS [12]		SNP [50]
*Dyrk1a*		De novo mutation [39]	
*Fmr1*	GWAS [15]		
*Gabrb3*	CNV [8]	SNP [57]	
*Grm3*	GWAS [12]		SNP [58]
*Katnal2*		De novo mutation [39]	
*Kctd13*	GWAS [12]		
*Kmt2c*	FMRP target [45, 46]	De novo mutation [59]	
*Kmt5b*		De novo mutation [39]	
*Kynu*	Exome sequencing [15]		
*Mecp2*	De novo mutation [60], FMRP target [45, 46]	De novo mutation [60]	
*Mib1*	miR137 target [14]		
*Nrxn1*	CNV [5], miR137 target [14], FMRP target [45, 46]		CNV [61]
*Pogz*		CNV [62], de novo mutation [39]	
*Pten*	miR137 target [14]		
*Reln*	SNP [63], FMRP target [45, 46]	SNP [64]	
*Scn2a*	miR137 target [14], FMRP target [45, 46]	De novo mutation [39]	
*Setd1a*	Exome sequencing [3]		
*Shank3*	CNV [9], FMRP target [45, 46]	CNV [65]	
*Stxbp1*	FMRP target [45, 46]		
*Stxbp5*	miR137 target [14]		
*Syngap1*	Exome sequencing [66], FMRP target [45, 46]	De novo mutation [39]	
*Tbr1*		De novo mutation [39]	
*Tcf4*	GWAS [12], miR137 target [14], FMRP target [45, 46]	SNP [40]	
*Top3b*	CNV [6]		
*Vipr2*	CNV [7]		

**Fig. 1:**
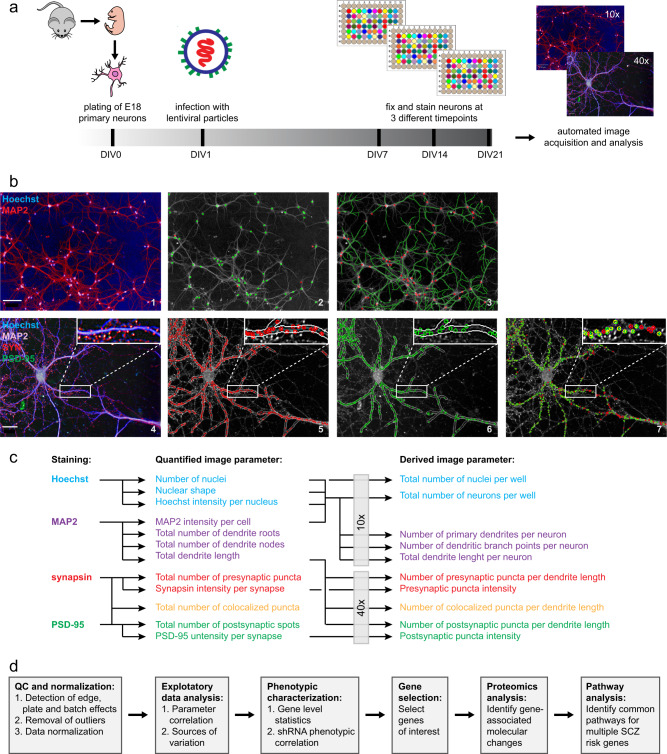
High-content screening and data analysis workflow. **a** E18 embryonic mouse neurons were seeded in 96-well cell culture plates and infected with lentiviral particles containing shRNA constructs against the 41 genes of interest (4–5 shRNAs per gene). Neurons were only cultured in the inner 60 wells of a 96-well plate and infected on day 1 in vitro (DIV1). On each plate, at least three wells were infected with a scrambled control shRNA, three wells with a positive control shRNA (against NLGN1), and at least three wells were left uninfected. The remaining wells were infected with ~ 50 different experimental shRNAs. Controls and experimental shRNAs were always in a randomized order as to minimize plate position effects. Cultures were fixed at DIV7, DIV14, or DIV21. For each time point, shRNA replicates (*n* = 3) were divided over three different culture plates as to minimize plate effects. **b** Neurons were stained with a nuclear marker (Hoechst), a dendritic marker (anti-MAP2), a presynaptic marker (anti-synapsin) and a postsynaptic marker (anti-PSD-95) and imaged using automated confocal high-content microscopy. Neurons were first imaged at × 10 magnification (panel 1) to determine neuron numbers (panel 2; MAP2-positive neurons in green; MAP2-negative cells in red), and total dendrite length and numbers of primary dendrites and branch points (panel 3; selected neurons in red, traced dendrites in green). Neurons were subsequently imaged at × 40 magnification (panel 4) to quantify presynaptic puncta (panel 5; selected puncta in red), postsynaptic puncta (panel 6; selected puncta in green), and colocalized pre-and postsynaptic puncta (panel 7; colocalized puncta in yellow). Images are representative examples of DIV14 neurons. Examples of neurons at all DIV are included in Supplementary Fig. S2. Scale bars: 100 μm (panels 1–3), 20 μm (panels 4–7). **c** Based on these primary measurements, 10 core parameters were derived that measure relevant aspects of neuronal viability and survival, neuronal network formation, and synaptic connectivity. **d** Data were checked for batch, plate, and edge effects and outliers were removed. After normalization, multi-level exploratory data analysis was performed to determine the relative contribution of both experimental and technical sources of variation to overall variance in the data. Statistical analysis was then performed to detect significant gene-level effects in the data without prior removal of individual shRNAs. In parallel, robust biological effects were determined by filtering out individual shRNAs that produce inconsistent phenotypes that might represent off-target effects. Based on these two selection criteria candidate genes were selected for proteomics analysis and subsequent cellular pathway analysis

For RNA interference screening, neurons were transduced with shRNA-containing LVV particles at day 1 in vitro (DIV1) and plates were fixed and stained at three time points (DIV7, DIV14, DIV21) to collect morphological data at different stages of neuronal network development (Fig. 1a). Neurons were stained for nuclei (Hoechst), dendrites (anti-MAP2), presynaptic puncta (anti-synapsin), and postsynaptic puncta (anti-PSD-95). Images were acquired using automated confocal microscopy and analyzed using automated image analysis software (Fig. 1b; Supplementary Fig. S2). Ten relevant measures of neuronal survival and network development were calculated (Fig. 1c) and a combination of data analysis tools and proteomics discovery experiments was used to identify neuronal phenotypes and molecular pathways of interest (Fig. 1d).

Inspection of plate heat maps revealed no major edge, plate, or batch effects on total cell numbers per well. When plotting all data per plate, one plate was a clear outlier with disproportionally high occurrence of low cell counts (Supplementary Fig. S3a), and these wells were removed. Data plots also revealed a consistent increase in total dendrite length per neuron and pre- and postsynaptic puncta densities with increasing DIV (Supplementary Fig. S3a), indicating that the image analysis algorithms reliably capture neuronal network maturation over time without saturation. To allow overall comparison, all data were normalized per plate against the median of the scrambled control and then log2 transformed. Untreated samples were found evenly distributed around zero, whereas shRNA-treated samples were skewed toward negative values, indicating that multiple shRNAs had negatively affected one or more neuronal network parameters (Supplementary Fig. S3b). Combining log-normalized data from all plates confirmed a normal distribution around zero for all 10 normalized and log2-transformed parameters with a slight skewedness toward negative values (Supplementary Fig. S4).

**Fig. 2:**
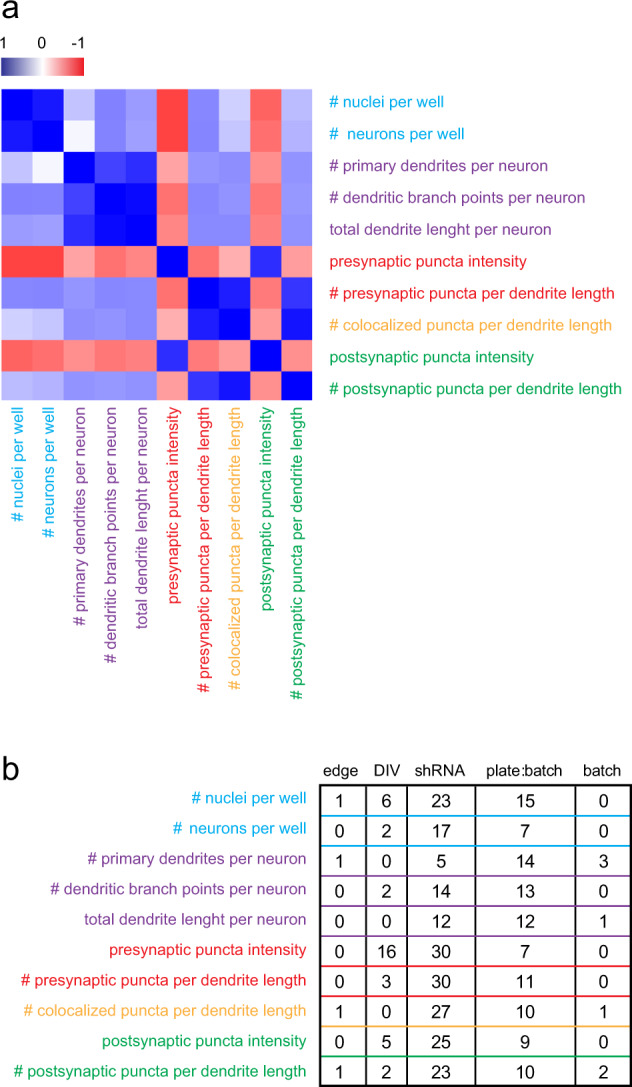
Exploratory analysis of normalized cellomics data. **a** Correlation analysis of 10 core parameters across all data reveals separation of cell number, dendritic, and synaptic parameter sets and strong correlations within each of these parameter sets. **b** Multi-level modeling was used to estimate variance in the data owing to technical variation (i.e., batch effects, plate-within-batch effects, and edge effects) or experimental variation (i.e., DIV and shRNA treatment). Normalized parameters are affected primarily by shRNA treatment, however, plate effects are observed for dendrite length, dendrite roots, and dendrite nodes. A complete overview of explained variances with confidence intervals is provided in Supplementary Table S3

Correlation analysis showed that the 10 selected parameters cluster into nuclear, dendritic, and synaptic parameter sets (Fig. 2a). Within each set, most parameters show a relatively strong positive correlation. Interestingly, pre- and postsynaptic intensities are negatively correlated with the other synaptic parameters (i.e., the intensities of synapsin and PSD-95 staining decrease as synapse numbers increase). Multi-level modeling was used to estimate the relative contribution of technical and experimental variation to overall variance in the data set, and we found that the experimental manipulation of shRNA treatment accounted for most of the parameter variance. Technical sources of variation had little effect (i.e., batch number, plate number within batch, edge location, and DIV). For dendritic parameters, relatively high variation was observed owig to plate-within-batch effects (Fig. 2b; Supplementary Table S3).

**Fig. 3:**
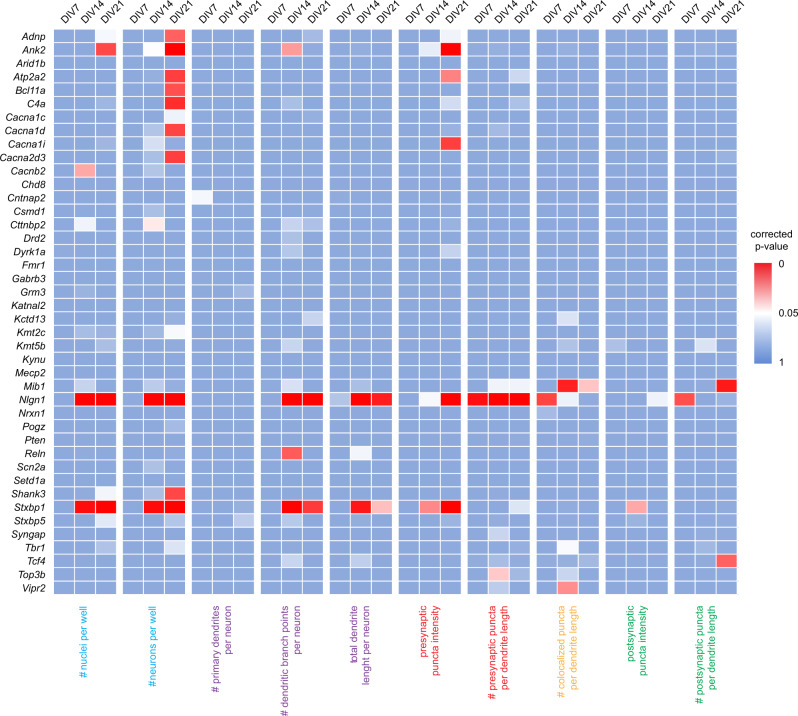
Statistical analysis of normalized data. A Mann–Whitney *U* test was performed per time point including all ten core parameters. Gene-level effects were calculated per parameter by averaging the normalized effects of all 4–5 gene-specific shRNAs. A permutation analysis was performed to correct for multiple testing. Heat maps show empirical *p* values for ten parameters across 41 genes at DIV7, DIV14, and DIV21

**Fig. 4:**
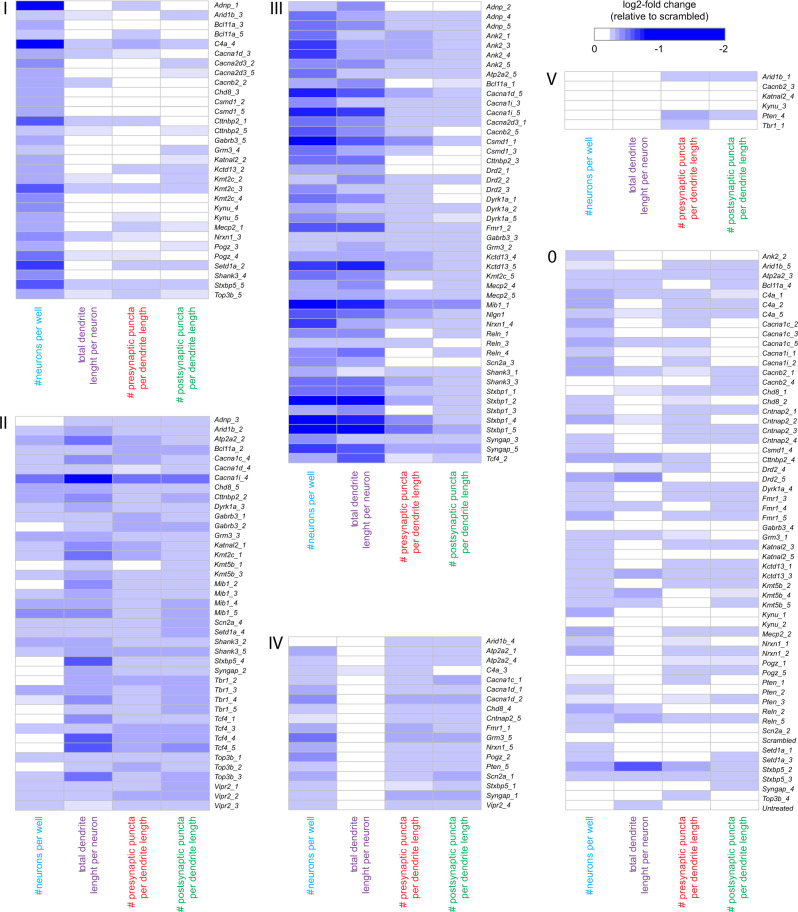
Phenotypic clustering of individual shRNAs. Hierarchical clustering was performed based on time point (DIV7, DIV14, and DIV21)-averaged data for four representative core parameters, total neuron number per well, total dendrite length per neuron, presynaptic puncta density, and postsynaptic puncta density. Before clustering, shRNAs with log-normalized effect sizes of ≤ 2 × the SD of the scrambled controls were excluded from the data and designated as the no-effect cluster (cluster “0”). For the remaining shRNAs, five phenotypic clusters were identified that are characterized by primarily a reduction in neuron numbers (cluster “I”), primarily a reduction in dendrite length and synapse densities (cluster “II”), an overall reduction in all parameters (cluster “III”), a reduction in synapse densities with little or no effect on other parameters (cluster “IV”), or the absence of a marked phenotype (cluster “V”)

**Fig. 5:**
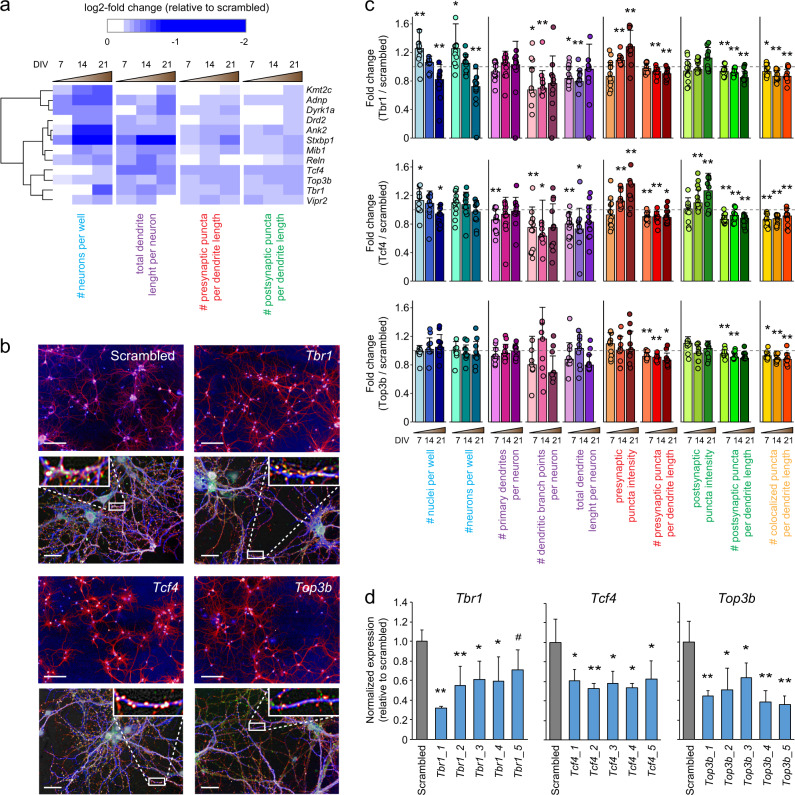
*Tcf4*−, *Tbr1*−, and *Top3b*-associated neuronal phenotypes. **a** Cluster analysis revealed 12 genes that show distinct and progressive neuronal phenotypes based on three or more co-clustering shRNAs per gene. *Tcf4*, *Tbr1*, and *Top3b* showed early or progressive synapse loss with a relatively small effect on neuron numbers. **b** Example images illustrate the neuronal morphology phenotypes of *Tcf4*, *Tbr1*, and *Top3b* knockdown cultures at DIV14 compared with a scrambled control culture. Scale bars: 100 μm (top panels), 20 μm (bottom panels). **c** Quantification of the *Tcf4*, *Tbr1*, and *Top3b* knockdown phenotypes across all 10 measured parameters demonstrates a significant reduction in pre-, post-, and/or colocalized synaptic puncta densities starting at DIV7, whereas neuronal cell numbers are not reduced until DIV21. Error bars represent SD; two-sided Student’s *t* test, *n* = 12 (*Tbr1* and *Tcf4*) or 9 (*Top3b*), *p < 0.05, **p < 0.01. **d** Quantitative real-time PCR shows that shRNAs targeting *Tcf4*, *Tbr1*, and *Top3b* reduce the corresponding mRNA levels by 30–70% relative to scrambled controls. Expression levels are normalized to the averaged expression levels of *Actb* and *Hprt*. Error bars represent SD; two-sided Student’s *t* test, *n* = 4, * *p* < 0.05, **p < 0.01, ^#^*p* < 0.01

To determine gene-level effects in the data, we first performed an unbiased statistical analysis of the averaged normalized values of all shRNAs per gene across all 10 parameters. Gene-level averaged parameter values were tested against the average of the scrambled control values for each time point separately. Statistically significant changes in any parameter other than “number of nuclei” or “number of neurons” were detected for *Ank2*, *Atp2a2*, *Cacna1i*, *Mib1*, *Nlgn1*, *Stxbp1*, *Tcf4*, *Top3b*, and *Vipr2* and (Mann–Whitney *U*, *p* < 0.05, corrected for multiple testing; Fig. 3). However, unbiased statistical analysis considers all shRNAs per gene as equally effective, which is unlikely and would result in underestimation of true biological effects. We therefore performed a cluster analysis to detect similarities in phenotypes across individual shRNAs and determined which shRNAs produce consistent, biologically relevant phenotypes. We selected four representative parameters: total number of neurons, total dendrite length per neuron, presynaptic puncta density, and postsynaptic puncta density. shRNAs with effect sizes less than twice the standard deviation of the scrambled controls for all four parameters at all three time points were excluded and were considered the no-effect group (Fig. 4; cluster “0”). Individual shRNAs were then clustered using a Pearson correlation distance matrix, resulting in five distinct clusters (Supplementary Fig. S5). These five clusters were characterized by: a reduction in neuron numbers (cluster “I”; 31 shRNAs); a reduction in dendrite length and synapse densities with little or no effect on neuron numbers (cluster “II”; 41 shRNAs); an overall reduction in all parameters (cluster “III”; 48 shRNAs); a reduction in synapse densities with little or no effect on other parameters (cluster “IV”; 18 shRNAs); and a small cluster with no effect across all four parameters (cluster “V”; 6 shRNAs) (Fig. 4). Twelve genes were identified with at least three shRNAs in the same phenotypic cluster: *Adnp*, *Ank2*, *Drd2*, *Dyrk1A*, *Kmt2c*, *Mib1*, *Reln*, *Stxbp1*, *Tbr1*, *Tcf4*, *Top3b*, and *Vipr2*. The co-clustering of three or more shRNAs for a single gene in the same phenotypic cluster was significantly different from chance (*χ*^2^ test, *p* = 1.6 × 10^−5^). When combining the effects of the co-clustered shRNAs per gene, the following phenotypes were identified: reduced neuron numbers (*Kmt2c*, *Adnp*, *Dyrk1a*), reduced dendrite length followed by a reduction in neuron numbers (*Drd2*, *Ank2*, *Stxbp1*, *Mib1*, *Reln*), and reduced dendrite length and synapse densities (*Tcf4*, *Top3b*, *Tbr1*, *Vipr2*) (Fig. 5a).

Given the polygenic nature of SCZ and the assumption that combined risk of many genes with small effect size is needed to develop SCZ, it is possible that risk genes whose knockdown produce similar cellular phenotypes converge on similar molecular pathways underlying those phenotypes. To test this hypothesis, we selected *Tcf4*, *Top3b*, and *Tbr1* as all showed a relatively strong and specific synaptic phenotype upon knockdown (Fig. 5a). Knockdown of all three genes resulted in a significant reduction of pre-, post-, and colocalized synaptic puncta densities starting at DIV7, whereas neuronal cell numbers were not reduced until DIV21 (Fig. 5b, c). Real-time quantitative PCR confirmed that all shRNAs producing the synaptic phenotype had knockdown efficiencies of 40–55% at DIV7, except for shRNA #5 targeting *Tbr1*, which was therefore excluded from further analysis (Fig. 5d). Noteworthy, the four shRNAs that did not cluster to the same phenotype (i.e., *Tcf4* #2, *Tbr1* #1, *Top3b* #4, and *Top3b* #5) did significantly reduce the corresponding mRNA levels. Phenotypically, *Tcf4* #2 and *Top3b* #5 showed in addition to a synapse loss also reduced cell viability, indicating potential off-target effects; *Tbr1* #1 and *Top3b* #4 showed a very mild synaptic phenotype but were classified as negatives, suggesting that they might actually be false negatives (Fig. 4).

To identify molecular pathways affected by *Tcf4*, *Tbr1*, or *Top3b*, we next performed a quantitative, label-free SWATH proteomics analysis of hippocampal cultures after knockdown of each gene with each shRNA separately. Proteomics analysis was performed at DIV7 when the synaptic phenotype was observed without significant changes in other cellular parameters. Peptide intensity distributions revealed high quality across all samples (Supplementary Fig. S6a, b) and, after setting optimal *q* value cutoffs for each experiment, we identified 2059 proteins in *Tbr1* knockdown samples, 2570 in *Tcf4* knockdown samples, and 2561 in *Top3b* knockdown samples (Supplementary Table S4). We next selected all proteins that showed a significant log2-fold change compared with scrambled shRNA-treated samples for at least one shRNA per gene (two-sided Student’s *t* test, *p* < 0.05) and a concordant change (i.e., in the same direction, but not necessarily statistically significant) for all other shRNAs targeting that gene. This analysis identified 94 consistently regulated proteins for *Tcf4*, 61 for *Tbr1*, and 67 for *Top3b* (Supplementary Fig. S6c**;** Supplementary Table S5). Only 2–5 proteins were regulated in common between any two genes, and only one protein between all three genes (Supplementary Fig. S6d).

**Fig. 6:**
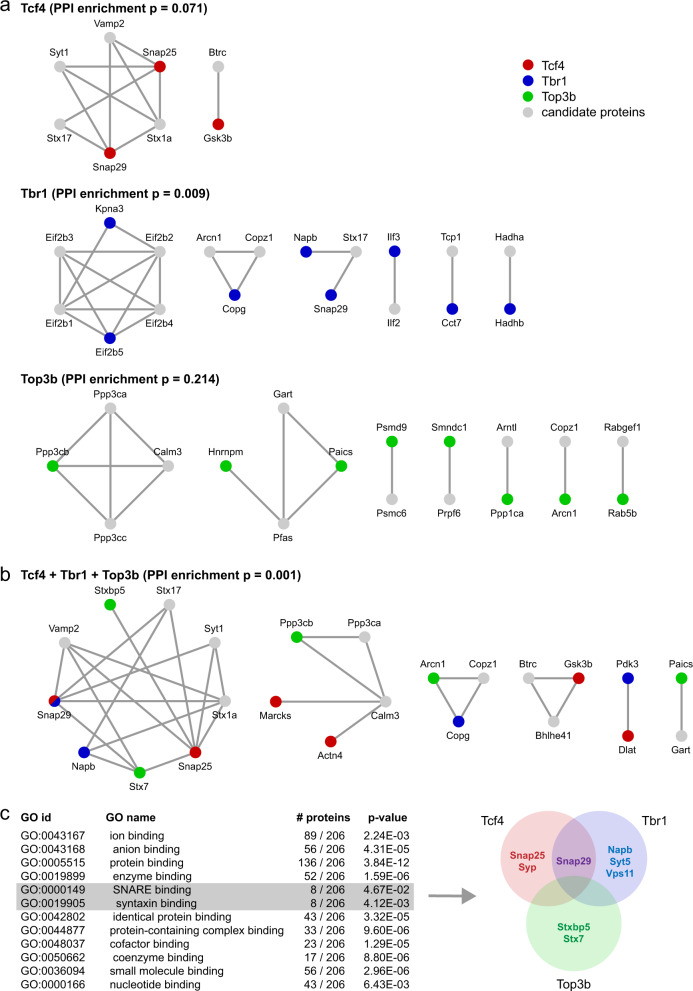
*Tcf4*−, *Tbr1*−, and *Top3b*-associated molecular pathways. **a** Proteomics analysis was used to identify dysregulated proteins owing to *Tcf4*, *Tbr1*, or *Top3b* knockdown. Dysregulated proteins were analyzed in STRING to identify PPI networks. Significant PPI enrichment was only detected in the *Tbr1* knockdown protein set. Red, blue, and green circles are query proteins that are dysregulated upon *Tcf4*, *Tbr1*, or *Top3b* knockdown, respectively, gray circles are candidate proteins added by STRING. **b** The same analysis was performed for the combined dysregulated protein set, identifying the SNARE complex as a potential common target of *Tcf4*, *Tbr1*, and *Top3b*. **c** GO enrichment analysis of the combined dysregulated protein set (without addition of candidate interactors) confirms enrichment for proteins involved in “SNARE binding” and “syntaxin binding” contributed by TCF4−, TBR1−, and TOP3B-dysregulated proteins

Protein–protein interaction (PPI) analysis was performed to predict cellular pathways that are dysregulated owing to either *Tcf4*, *Tbr1*, or *Top3b* knockdown. High-confidence PPI networks were extracted from STRING [22] based on experimentally validated interactions only using and allowing the addition of maximally 10 network candidate proteins. Significant PPI enrichment was detected in the TBR1-regulated protein set (*p* = 0.009), but not in the TCF4- or TOP3B-regulated protein sets (*p* = 0.071 and *p* = 0.214, respectively), and no detected network contained more than two regulated proteins (Fig. 6a). To identify PPI networks that may be dysregulated owing to the combined effect of *Tcf4*, *Tbr1*, and *Top3b* knockdown we also performed an analysis of all 210 regulated proteins together. PPI enrichment in this combined protein set was highly significant (*p* = 0.001), and the largest PPI network that was identified consisted of SNARE proteins involved in neurotransmitter release and contained five dysregulated proteins (SNAP25, SNAP29, NAPB, STX7, and STXBP5) from all three data sets (Fig. 6b). Smaller networks contained no more than three proteins from maximally two data sets.

To exclude the possibility that detection of the SNARE complex was owing to the addition of network candidates that are not part of the dysregulated protein set we also performed gene ontology (GO) enrichment analysis of dysregulated proteins only using gProfiler [23]. Significant enrichment was observed for “molecular function” GO classes “SNARE binding” (*p* = 0.047) and “syntaxin binding” (*p* = 0.004), and three proteins were identified (SYP, SYT5, and VPS11) in addition to the five found previously using PPI analysis (Fig. 6c). PPI and GO enrichment analysis together thus strongly suggest that reduced expression of SCZ risk genes *Tcf4*, *Tbr1*, and *Top3b* converges on a canonical neurotransmitter release pathway involving the SNARE protein complex.

## Discussion

SCZ is a highly complex polygenic disorder for which > 145 genomic regions have been significantly associated in genetic studies [12, 13]. This poses the challenging question how this genetic variation converges onto common patterns of cellular and molecular alterations underlying the disorder. In-depth studies of individual risk genes, isolated from a spectrum of hundreds, have provided limited information on disease biology. In this study, we integrated cellomics and proteomics approaches to demonstrate that significant insight into disease biology can be obtained by combining neuronal cellular morphology and molecular pathway information for multiple risk genes and searching for shared characteristics.

RNA interference and subsequent high-content neuronal cellular phenotyping of 41 candidate SCZ risk genes resulted in the identification 12 genes showing robust phenotypic clustering and producing reliable phenotypes with multiple shRNAs targeting the same gene. Based on these findings, and assuming that polygenic risk is essential in SCZ, we hypothesized that SCZ risk genes that share a similar neuronal phenotype may each contribute to that phenotype in a synergistic manner. To test this, we selected *Tbr1*, *Tcf4*, and *Top3b*, which all showed a common knockdown phenotype characterized by a reduction in synapse densities, in line with the suggested developmental nature of SCZ [24]. Importantly, reduced synapse densities were observed early, before neuron numbers became affected, indicating that synaptic connectivity impairments were driving neuronal loss, and not the other way around. All three genes regulate gene expression, but none have been functionally linked yet. TBR1 is a T-box transcription factor involved in the development of cortical and amygdala neurons [25, 26] and impairments in hippocampal neurogenesis were shown to correlate with reduced *TBR1* expression in an induced pluripotent stem cells (iPSC) model of SCZ [27]. TBR1 was shown to induce the expression of NMDA receptor subunit *Grin2b* in hippocampal and amygdala neurons in an activity-dependent manner [28]. TCF4 is a basic helix–loop–helix transcription factor previously associated with SCZ, ASD, and intellectual disability [29] and has been shown to regulate spine densities in the cortex and the hippocampus [30]. TOP3B is a topoisomerase that associates with FMRP in a direct, non-mRNA-mediated manner [6]. FMRP regulates the translation of synaptic mRNAs and the development and function of synapses [31], and genes encoding FMRP target mRNAs are significantly enriched for SCZ-associated rare mutations [15]. It is not clear whether TBR1, TCF4, and TOP3B regulate synaptic functions independently, or if they converge on shared synaptic pathways.

Our proteomics experiments and subsequent PPI and pathway analyses highlight syntaxin-mediated neurotransmitter release as a prominent pathway onto which TCF4-, TBR1-, and TOP3B-regulated protein expression converges. Central in this network is syntaxin 1A (STX1A), which is crucial for synaptic vesicle docking and neurotransmitter release [32]. A genetic association between *STX1A* and SCZ has been suggested [33] and STX1A mRNA and protein levels are decreased in post-mortem brain tissue from SCZ patients [34]. Importantly, STX1A itself was not dysregulated in our proteomics experiments. Instead, multiple nodes in the STX1A PPI network were affected by *Tcf4*, *Tbr1*, or *Top3b* knockdown. Other genes that were included in the screen may impact on the same pathway. *Stxbp1* for instance is essential for syntaxin-mediated neurotransmitter release [35], and its knockdown resulted in a much stronger phenotype than *Tcf4*, *Tbr1*, or *Top3b* knockdown, including also reduced dendrite length and reduced neuronal viability. This suggests that neurotransmitter release in SCZ may be affected owing to subtle dysregulation via indirect upstream gene regulatory mechanisms rather than a dysregulation of proteins that are directly involved. In line with this, we could not find other SZC GWAS candidate genes that are part of the same neurotransmitter release pathway, nor did we observe significant enrichment for low *p* value GWAS hits among all proteins dysregulated by *Tcf4*, *Tbr1*, or *Top3b* knockdown (*p* = 0.44, 0.48, and 0.91, respectively; hypergeometric test). Together, these observations demonstrate that interpreting polygenic risk from a protein regulatory network perspective is able to uncover hidden aspects of disease biology.

Our study provides proof-of-principle that multi-level morphological and molecular phenotyping is able to extract disease-relevant pathway information about neuropsychiatric disorders, however, several limitations apply. For instance, stringent selection criteria (i.e., three or more shRNAs against the same gene producing a similar phenotype) led to the identification of only 12 candidate genes for follow up analysis. Although this prevented potential off-target effects from biasing the data towards false positive hits, it likely also increased false negative findings. Interestingly, another 10 genes (*Atp2a*, *Cacna1d*, *Cacna1i*, *Csmd1*, *Gabrb3*, *Kctd13*, *Kmt5b*, *Mecp2*, *Shank3*, and *Syngap*) were assigned with two shRNAs to one of the three phenotypic clusters marked by synaptic changes (i.e., clusters II, III, or IV in Fig. 4), including candidate synapse- or neurotransmission-modifying genes such as *Cacna1d*, *Cacna1i*, *Gabrb3*, *Mecp2*, *Shank3*, and *Syngap*. Likewise, additional pathways may be revealed when proteomics profiling is performed for more candidate genes in every phenotypic cluster. In particular, our screen included, in addition to *Tcf4*, *Tbr1*, or *Top3b*, nine more transcriptional regulators and chromatin modifiers that produced dendritic or synaptic changes (i.e., *Adnp*, *Arid1b*, *Bcl11a*, *Chd8*, *Kmt2c*, *Kmt5b*, *Mecp2*, *Pogz*, and *Setd1a* in clusters II, III of IV; Fig. 4). These are interesting candidate genes to discover additional converging pathways leading to neuronal connectivity changes underlying SCZ.

In this study, we used mouse primary neuronal cultures combined with RNA interference to study SCZ risk gene function. Given the fact that SCZ is a complex human disease and the underlying genetic variation cannot simply be modeled by reducing the expression of individual genes, this is a strongly reductionist approach. It is however unlikely that patient mutations that individually do not increase SCZ risk would produce detectable phenotypes in a cellular assay. RNA interference on the other hand allows functional characterization and clustering of candidate SCZ risk genes based on detectable phenotypes, and permits subsequent identification of shared molecular and cellular pathways. Our data show that using this approach, disease-relevant pathway information can be obtained and used to generate testable hypotheses with respect to multi-gene interactions that increase SCZ risk in humans. These hypotheses can then be tested in other models, for instance iPSC-derived human neurons, in which mutations that are predicted to converge onto common cellular pathways co-exist, either by nature or engineered.

It is still an open question whether genetic risk for SCZ converges onto one or more common cellular pathways. The fact that three non-synaptic genes show downstream convergence onto an important synaptic signaling pathway argues that the disease may be more homogeneous than suggested by its apparent genetic complexity, and is consistent with previous speculations based on genetic findings that synapses are an important substrate in SCZ pathology [36–38]. Another issue of debate is the genetic similarity between SCZ and other psychiatric disorders, in particular BPD and ASD. *TBR1* has also been associated with ASD [39] and *TCF4* with BPD [40], and TOP3B was shown to bind multiple mRNAs derived from ASD-linked genes [41], raising the possibility that STX1A-mediated neurotransmitter release is dysregulated in multiple psychiatric disorders. Indeed, genetic or functional associations exist between STX1A and BPD [42] or ASD [43]. Pathway analysis of additional risk genes may strengthen the hypothesis that neurotransmitter release pathways are common substrates for multiple psychiatric disorders.

## Supplementary information

Supplementary Figures S1-S6

Supplementary Table S1

Supplementary Table S2

Supplementary Table S3

Supplementary Table S4

Supplementary Table S5
