# Supplementary material for: Combined cellomics and proteomics analysis reveals shared neuronal morphology and molecular pathway phenotypes for multiple schizophrenia risk genes
Source: Mol Psychiatry. 2019 May 29;26(3):784–99. doi: 10.1038/s41380-019-0436-y (PMC7910218; doi:10.1038/s41380-019-0436-y)
Supplement: Supplementary file 1 — Supplementary Figures S1-S6 [file 41380_2019_436_MOESM1_ESM.pdf]

A

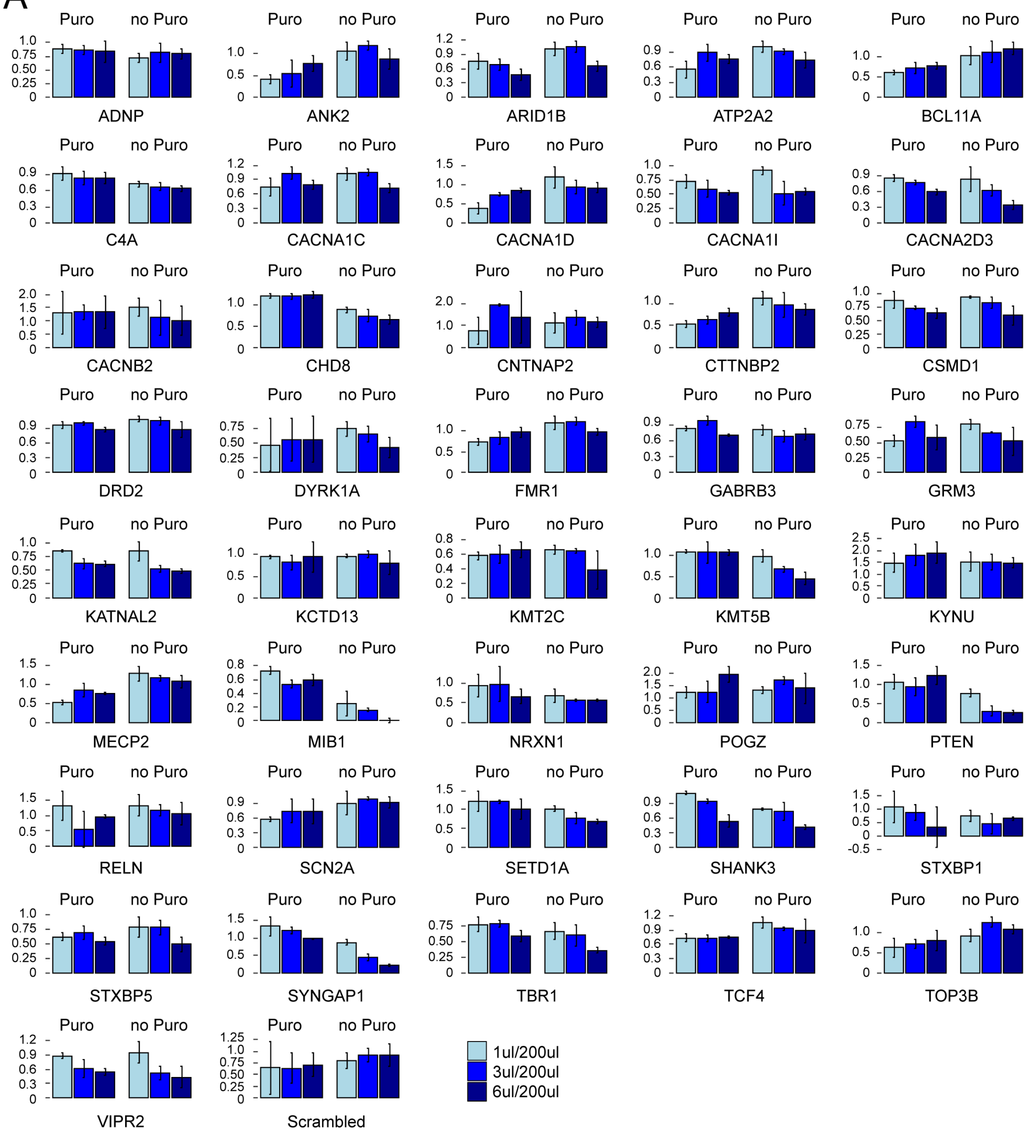

B

Puromycin	> 0.7	> 0.8	> 0.9
1ul/200ul	68%	49%	34%
3ul/200ul	70%	51%	31%
6ul/200ul	66%	41%	29%

no Puromycin	> 0.7	> 0.8	> 0.9
1ul/200ul	92%	73%	56%
3ul/200ul	65%	53%	48%
6ul/200ul	51%	41%	29%

Puromycin	Median	St Dev
1ul/200ul	80%	28%

no Puromycin	Median	St Dev
1ul/200ul	94%	24%

Supplementary Figure S1. Titration of optimal virus concentrations. (**a**) The effects of shRNA transduction on neuronal survival and viability were tested for 41 shRNAs, one shRNA per gene, at 3 virus concentrations (1, 3 and 6 μl/200 μl medium). Neurons were infected at DIV1 and cultured in the absence or presence of puromycin (0.2 μg/μl, added at DIV2). Neurons were fixed and stained at DIV7 (puromycin treated) or DIV14 (untreated). Bar graphs show viable neuron counts per well, normalized against untreated (no shRNA, no puromycin) controls (*n* = 3). (**b**) Quantification of the fraction of shRNAs resulting in either 70%, 80% or 90% viable neurons showed no increase in neuronal survival when increasing virus concentrations from 1 μl/200 μl to 6 μl/200 μl medium in the presence of puromycin, indicating no overall increase in infection efficiency. In the absence of puromycin however, 70% viability numbers dropped from 92% to 51% with increasing virus concentrations, indicating an overall increase in general shRNA-induced toxicity. Based on these findings we selected 1 μl/200 μl medium as the optimal virus concentration for all shRNAs. On average, this virus concentration produced an infection efficiency of 80% ± 28%.

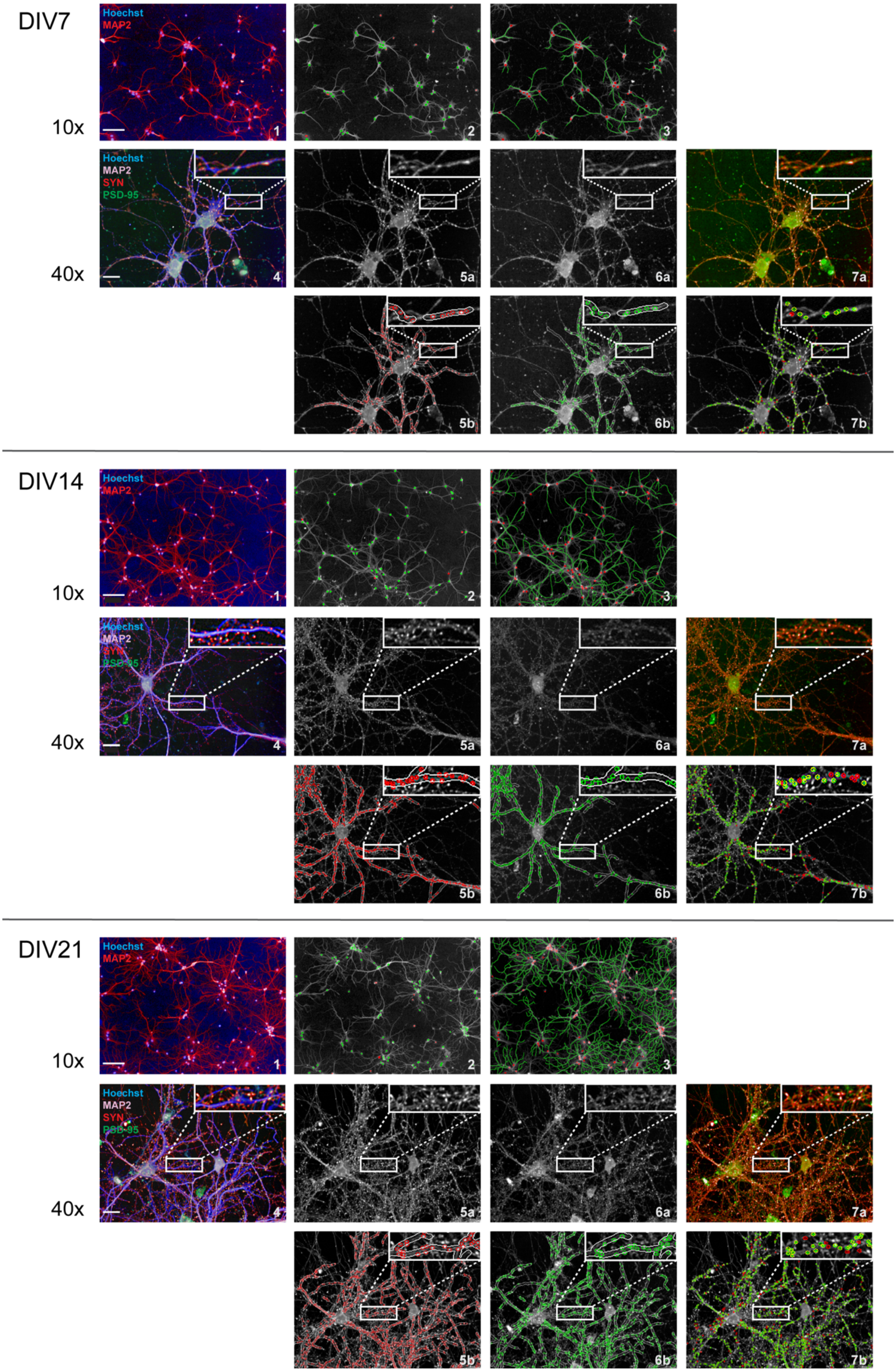

Supplementary Figure S2. Automated image analysis of cultured hippocampal primary neurons at DIV7, DIV14 and DIV21. Neurons were stained with a nuclear marker (Hoechst), a dendritic marker (anti-MAP2), a presynaptic marker (anti-synapsin) and a postsynaptic marker (anti-PSD-95) and imaged

using automated confocal high-content microscopy. Neurons were first imaged at 10x magnification (panel 1) to determine neuron numbers (panel 2; MAP2-positive neurons in green; MAP2-negative cells in red), and total dendrite length and numbers of primary dendrites and branch points (panel 3; selected neurons in red, traced dendrites in green). Neurons were subsequently imaged at 40x magnification (panel 4) to quantify presynaptic puncta (panel 5a,b; selected puncta in red), postsynaptic puncta (panel 6a,b; selected puncta in green) and colocalized pre-and postsynaptic puncta (panel 7a,b; colocalized puncta in yellow). Scale bars: 100 μm (panels 1-3), 20 μm (panels 4-7).

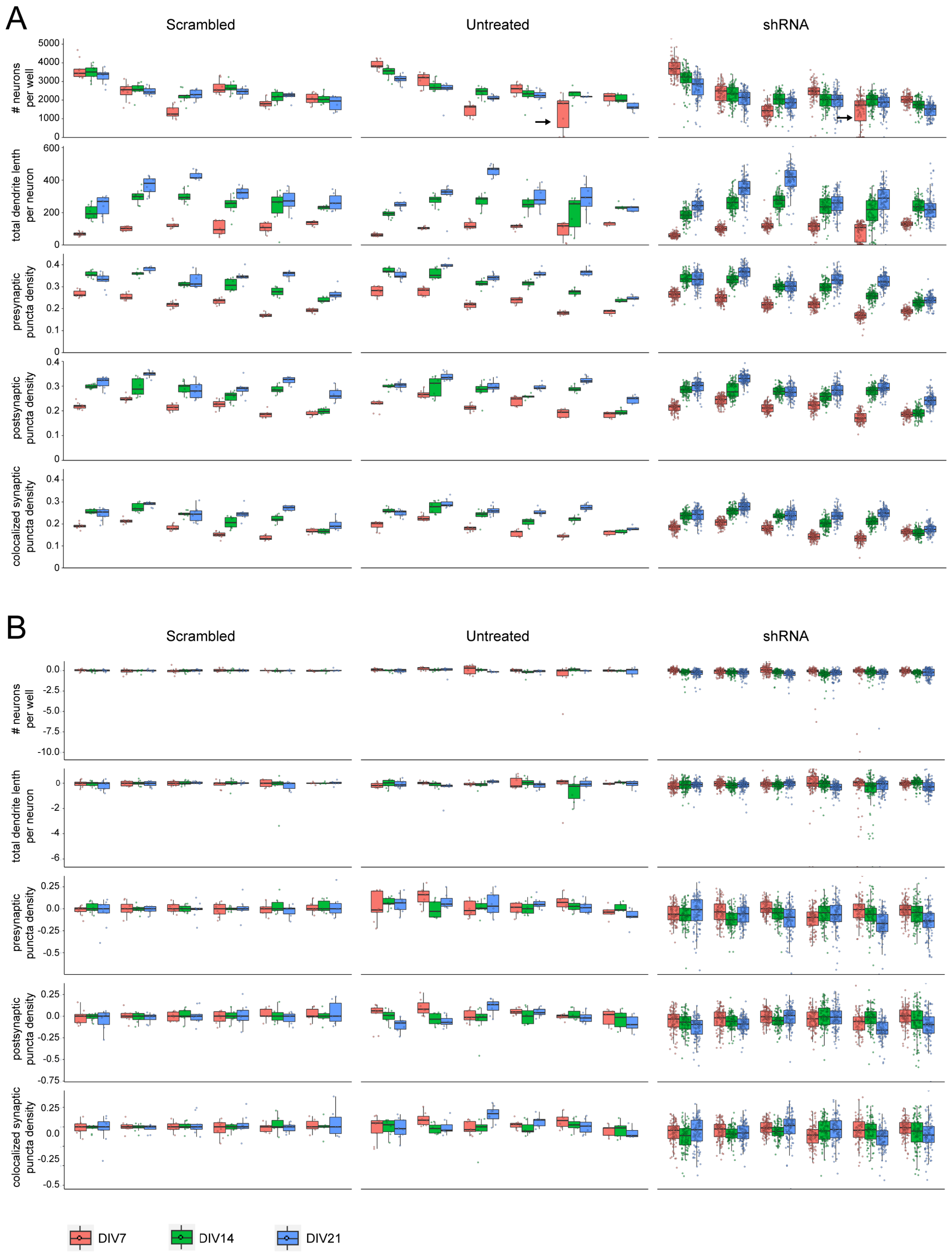

Supplementary Figure S3. Exploratory analysis of non-normalized cellomics data. (**a**) Box plots show the distributions of non-normalized parameters *total neuron number per well*, *total dendrite length per neuron*, *presynaptic puncta density*, *postsynaptic puncta density* and *colocalized synaptic puncta density* per plate, grouped per DIV and per batch. Scrambled and untreated controls are plotted separately from experimental shRNAs. Some plate and batch variability can be observed in all parameters, reflecting experimental variation due to differences in input cell numbers and culture conditions. At the same time, dendrite length and synapse densities increase with increasing DIV,

indicating that these parameters reliably measure network maturation. One outlier plate (indicated with arrow) showed significant cell death on one half of the plate, and the affected wells were removed from further analysis. (**b**) After outlier removal, per plate normalization against scrambled controls and log2 transformation, data were comparably between plates and batches, and show increasing variation with DIV due to gene-specific shRNA effects that increase over time.

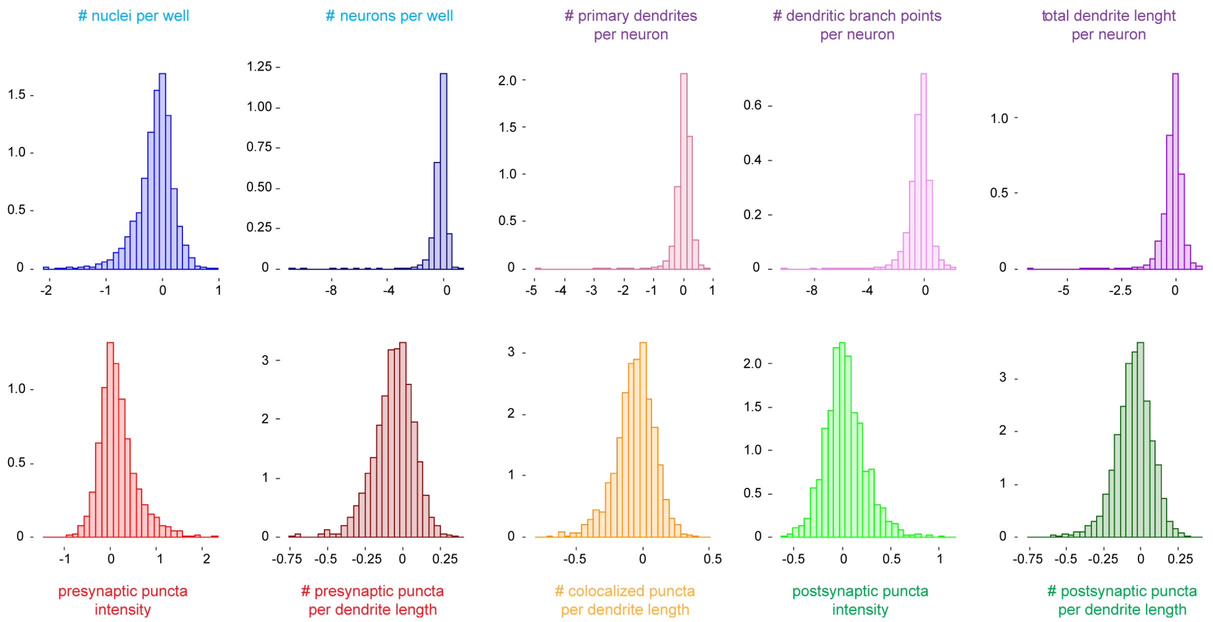

Supplementary Figure S4. Distribution of normalized cellomics data. (**a**) All log-normalized cellomics parameters are normally distributed around zero across all data.

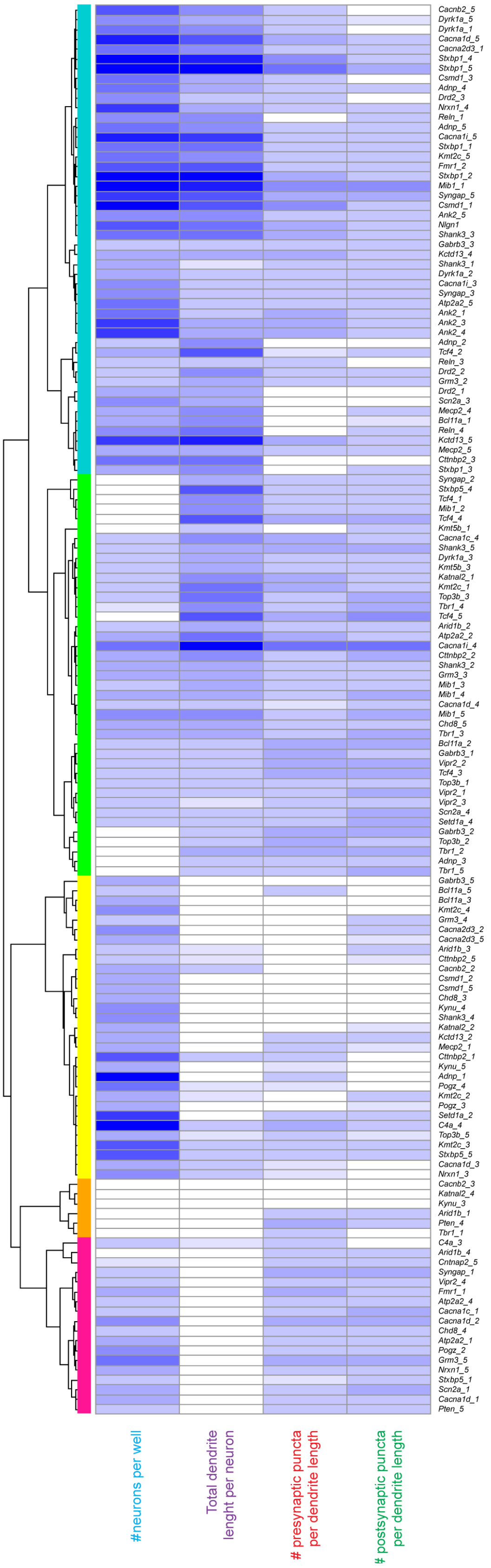

Supplementary Figure S5. Cluster tree of individual shRNA phenotypes. After removal of shRNAs with no phenotypic effect (log-normalized effect sizes of ≤ 2x the SD of the scrambled controls), the remaining shRNAs were clustered according to their phenotype. The cluster tree was then divided into 5 separate phenotypic clusters according to the colors in the vertical bar.

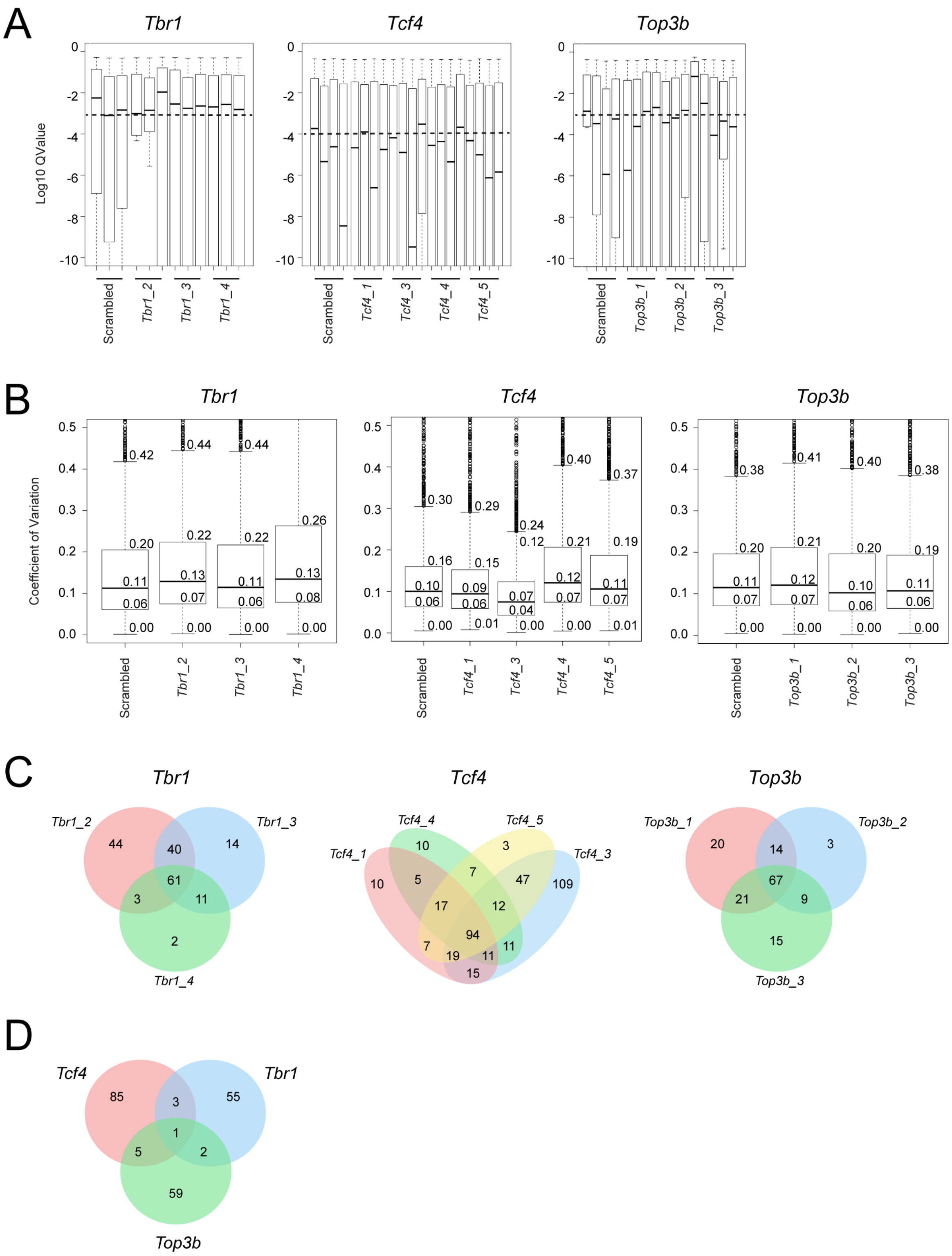

Supplementary Figure S6. Proteomics analysis of *Tcf4*, *Tbr1* and *Top3b* knockdown cultures. (**a**) Bar graphs show peptide Q-value distributions of all replicate samples (*n* = 3 per shRNA). Peptides with unreliable identification were cut off at 10^{-3} (*Tbr1* and *Top3b*) or 10^{-4} (*Tcf4*). (**b**) Bar graphs show distributions of CV-values per shRNA. Median CV-values of <0.15 indicate high quality data. (**c**) Venn diagrams show overlap in regulated proteins (compared with scrambled controls) for individual shRNAs per gene. (**d**) Venn diagram shows overlap in regulated proteins (compared with scrambled controls) for each gene.
